# Transthoracic echocardiography-guided PBMV for severe rheumatic mitral stenosis with pregnancy

**DOI:** 10.1093/qjmed/hcae046

**Published:** 2024-04-05

**Authors:** Hao Xiao, Pan Xiangbin, Hu Haibo

**Affiliations:** Cardiovascular Department Division 1, Hebei General Hospital, 348 Heping West Road, Hebei, Shijiazhuang 050000, China; Center of Structural Heart Disease, State Key Laboratory of Cardiovascular Disease, Fuwai Hospital, National Center for Cardiovascular Diseases, Chinese Academy of Medical Sciences and Peking Union Medical College, Beilishi Road, Xicheng District, Beijing 100037, China; Center of Structural Heart Disease, State Key Laboratory of Cardiovascular Disease, Fuwai Hospital, National Center for Cardiovascular Diseases, Chinese Academy of Medical Sciences and Peking Union Medical College, Beilishi Road, Xicheng District, Beijing 100037, China

Learning points for clinicians (i) Younger patients are suitable for percutaneous balloon mitral valvuloplasty; (ii) as a guiding method for interventional procedures, transthoracic echocardiography is suitable for PBMV in some special patients; and (iii) with the advancement of ultrasound technology, interventional procedures guided by transthoracic echocardiography will be increasingly implemented.

## Background

The currently invasive strategies for mitral valve stenosis include percutaneous balloon mitral valvuloplasty (PBMV) and surgical replacement. Most PBMV procedures are guided by fluoroscopy.^1–3^ For some patients, such as pregnancy, the use of radiation may have an impact on the fetus. We report a case of PBMV guided by transthoracic-echocardiography.

## Case summary

The patient is a 29-year-old female with 15 weeks of pregnancy. She experienced shortness of breath for 1 month after physical activity, accompanied by decreased activity endurance. Pre-procedure examination of the apex of the heart revealed open valve sounds and 4/6 level diastolic murmurs accompanied by tremors, radiating to the left axilla. Pre-procedure transthoracic and transesophageal echocardiography showed thickening of mitral valve leaflets, enhanced echogenicity, and mild calcification, resulting in restricted opening. Wilkins scored 6 points.

**Figure 1.**
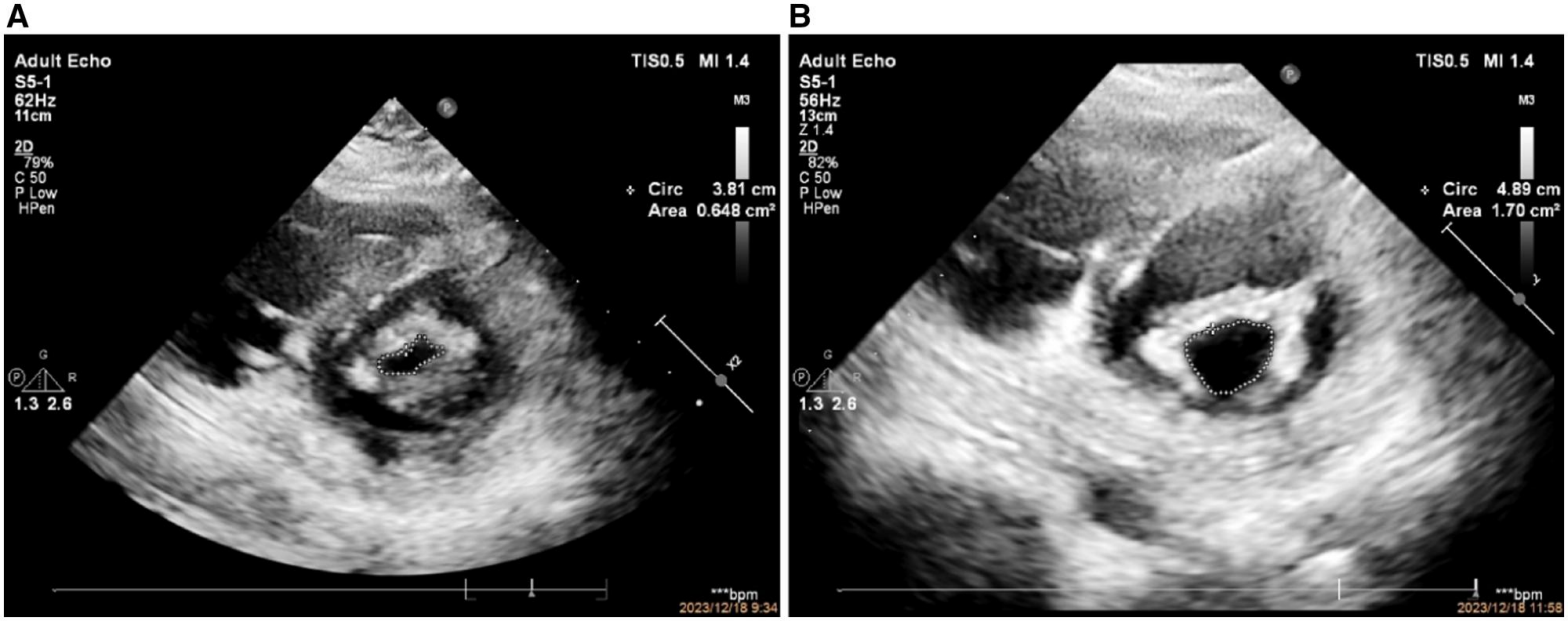
Pre-procedure and post-procedure mitral valve diastolic valve opening area (**A**, a pre-procedure mitral valve opening area of 0.648 cm^2^ during diastole; **B**, post-procedure mitral valve diastolic valve opening area of 1.7 cm^2^).

**Figure 2.**
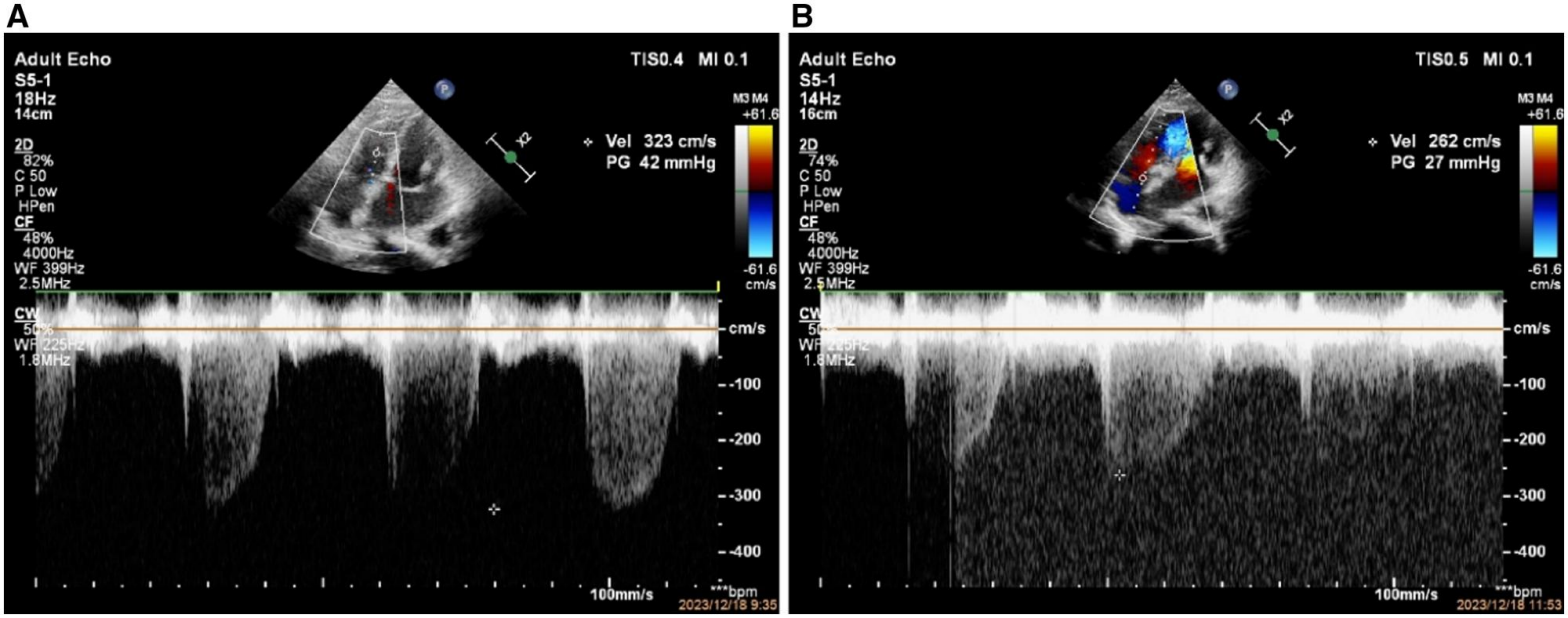
Pre-procedure and post-procedure tricuspid regurgitation velocity and estimated pulmonary artery systolic pressure (**A**, pre-procedure tricuspid regurgitation velocity 3.23 m/s, estimated pulmonary artery systolic pressure 42 mmHg; **B**, post-procedure tricuspid regurgitation velocity 2.62 m/s, estimated pulmonary artery systolic pressure 27 mmHg).

**Figure 3.**
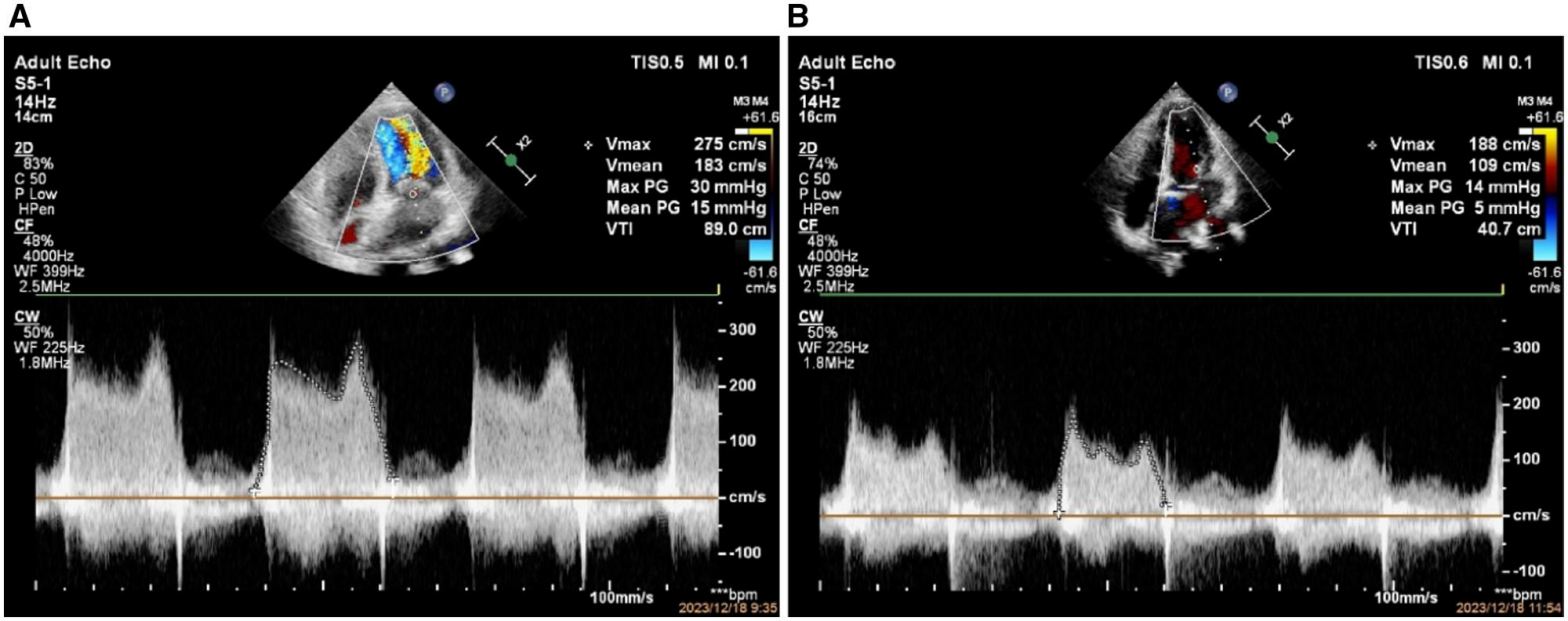
Pre-procedure and post-procedure mitral valve spectrum measurements (**A**, pre-procedure average mitral valve velocity of 1.83 m/s and average transvalvular gradients of 15 mmHg; **B**, post-procedure average mitral valve velocity of 1.09 m/s and average transvalvular gradients of 5 mmHg).

Due to pregnancy, we consider a procedure guided by transthoracic echocardiography (TTE) under local anesthesia. We chose a 26-mm Inoue balloon (with a diameter of 25 mm after water-injection) for PBMV procedure. After dilation, transthoracic-ultrasound showed improvement in the opening of the mitral valve, with a valve opening area of 1.7 cm^2^ from 0.64-cm^2^ pre-procedure (Figure 1). Pulmonary artery pressure decreased significantly (Figure 2). Mean left atrial pressure was measured at 10 mmHg after dilation (pre-procedure pressure is 39 mmHg), and there was no severe mitral regurgitation (Figure 3). Post-procedure auscultation revealed 2/6 grade rumbling murmurs in the apex area, without tremors or radiation.

## Discussion

X-ray-guided PBMV increases the risk of treatment, including X-ray glandular injury, fetal injury, contrast agent allergy, and contrast agent nephropathy. Previous studies have found that ultrasound-guided PBMV, percutaneous closure of atrial septal defects, ventricular septal defects, and patent foramen ovale have all achieved good results.^4^ In this case, PBMV of mitral valve stenosis under pure ultrasound guidance was more effective than double guidance with X-ray and ultrasound. Both methods significantly reduced transvalvular gradient differences and increased the valve opening area. However, pure ultrasound guidance reduced radiation-related injuries to medical staff and patients.^5^ Therefore, pure ultrasound guidance,^6^ especially PBMV guided by TTE, reduces radiation-related damage and can be the preferred guidance method for PBMV.

## Statements

(i) The investigation conforms to the principles outlined in the Declaration of Helsinki. (ii) Before the PBMV procedure, we have informed patients and families of the relevant risks and obtained information on their complications; at the same time, we also obtained informed consent from patients regarding the use of their samples. (iii) All operations comply with clinical practice and local ethical standards.
